# Characterizing Refractive Errors, Near Accommodative and Vergence Anomalies and Symptoms in an Optometry Clinic

**DOI:** 10.22599/bioj.267

**Published:** 2022-07-14

**Authors:** Samuel O. Wajuihian

**Affiliations:** 1 University of Kwazulu-Natal School of Health Sciences, ZA

**Keywords:** accommodative anomalies, vergence anomalies, optometry clinic, symptoms, students and young adults

## Abstract

**Background:**

Refractive, accommodative and vergence parameters and associated anomalies cause symptoms of asthenopia. Patients consult eye care practitioners mainly due to symptoms they experience. To enhance targeted treatments from various anomalies, it is relevant to study symptoms with associating anomalies.

**Aim:**

To determine the frequencies of refractive error, accommodative and vergence anomalies, and their associations with symptoms in sample of Black South Africans.

**Method:**

This prospective, cross-sectional study comprised consecutive participants aged 10–40 years who attended the author’s optometry practice in a Black population in South Africa. Visual acuity, refraction, accommodative and vergence tests were performed. Anomalies were classified as either single measure or syndromes based on the number of failed clinical signs.

**Results:**

Participants (n = 254) had mean age 22.6 ± 7.22 years. Ninety-four were male (37%) and 160 were female (63%). The frequencies of syndrome anomalies were accommodative insufficiency 17 [(6.6%) 95% CI 3.9–10.5%)], accommodative infacility 32 [(12.6%)] 8.7–17.3%] and convergence insufficiency 22 [(8.6%, 5.1–12.3%)]. Frequencies of coexisting anomalies were refractive error and accommodative 150 (60.0%), refractive error and vergence anomalies 136 (54.4%) and vergence and accommodative disorders 155 (62.0%). Most patients were symptomatic (70.9%). Headache was the most frequent symptom (41.1%).

**Conclusion:**

Accommodative anomalies were more frequent than refractive error and vergence anomalies. The high frequency of anomalies suggests a high uptake of optometric services for asthenopia. Accommodative anomalies were the most symptomatic. The study highlights the need for diagnosing visual symptoms and coexisting anomalies. Establishment of validated study protocols for all accommodative and vergence anomalies is recommended.

## Introduction

Refractive errors, as well as non-strabismic accommodative and vergence binocular comprise the visual efficiency system and a clear and comfortable vision is attained when the visual efficiency mechanism is optimum (Garzia 2006; Scheiman & Wick 2014). Patients’ refractive status relate to potential accommodative and vergence anomalies in both aetiology and treatment. When visual efficiency anomalies coexist, the conventional principle of maximum plus and minmum minus corrections may not apply (Dwyer & Wick 1995; Ma et al. 2019). Similary, when there is anticipation that an esophoric patient’s symptoms would resolve with hyperopic correction or myopic correction for exophoria may also not apply. For both instances, the dynamics and nature of coexisting anomalies should be considered before treatment is initiated to enhance utmost prognosis.

Anomalies of the visual efficiency system and associated symptoms affect clarity, comfort, binocularity, and impair efficiency of visual performance of an individual when near tasks are performed (Gall & Wick 2003; Garzia 2006). The similarities of symptoms among visual efficiency anomalies is a concern for clinical practice as each anomaly may require treatment approaches. Thus, in most cases, when symptoms are similar in diagnosed visual efficiency anomalies, differential diagnosis is warranted. Studying anomalies – including those coexisting and associated symptoms – to enhance understanding is thus relevant.

Given the outlined challenges, the negative impact of symptoms on performance and quality of life of individuals, researchers have studied various aspects of visual efficiency anomalies and symptoms in order to enhance understanding. Therefore, here in an abridged review of previous studies, only the frequency ranges are presented. For clinical studies on vergence anomalies (syndromes) in school aged and young adult populations, the frequency of convergence insufficieny (CI) ranged between 0.8 and 32% (Daum 1984; Hokoda 1985; Lara et al. 2001; Ma et al. 2019; Magdelene et al. 2017; Montes Mico 2001; Ovenseri-Ogbomo & Eguegu 2016; Rouse et al. 1998) convergence excess (CE) ranged between 0.6 and 9.2% (Daum 1984; Ma et al. 2019; Magdelene et al. 2017; Mandal & Kamath 2020; Moon, Kim & Yu 2020; Ovenseri-Ogbomo & Eguegu 2016) while fusional vergence dysfunction (FVD) ranged between 1.5 and 2.7% (Ma et al. 2019; Montes Mico 2001; Richman & Laudon 2002). For accommdative anomalies (syndromes), the frequency of accommodative insufficieny (AI) ranged between 2.4 and 41.3% % (Daum 1984; Hokoda 1985; Ma et al. 2019; Lara et al. 2001; Magdelene et al. 2017; Montes Mico 2001; Ngakhushi et al. 2018) while accommodative infacility (AIF) ranged betwee 2.1 and 40% (Hokoda 1995; Ma et al. 2019; Montes Mico 2001; Ngakhushi et al. 2018). For single clinical measure anomalies, the overall vergence anomalies ranged between 38 and 58% (Dwyer 1992; Montes-Mico 2001) while accommodative anomalies ranged between 57 and 62% (Dwyer 1992; Hoffman & Cohen 1973; Montes-Mico 2001). Regarding symptoms, the design of questionnaires differed across studies; however, a consistent finding from previous studies is that the frequencies of asthenopia are high and headache is the most frequent symptom among patients who consulted the optometrists, with a frequency range between 11.6% and 93%.

The review of previous studies revealed that only one available study, by Ovenseri-Ogbomo & Eguegu (2016) in Nigeria, reported on aspects of visual efficiency anomalies among Black African population. The frequencies of coexisting anomalies were rarely studied, and this limitation was highlighted by Dwyer (1996), who noted that accommodative and vergence anomalies commonly coexisted in clinical practice but are often not researched. Another limitation is that several studies (Daum 1984; Hokoda 1985; Mandal & Kamath 2020; McKay, Woodruff & Rumsey 2002; Moodley 2008; Rouse et al. 1998) applied retrospective design (Paniccia &Ayala 2015; Rouse et al. 1998). Furthermore, the criteria applied to define anomalies in most studies were derived from populations which may be inappropriate for their study sample and populations. On the relation of symptoms to anomalies, most available studies investigated only limited anomalies in relation to symptoms and only Dwyer (1992) investigated refractive error and accommodative and vergence anomalies in relation to asthenopia in a clinical optometry setting.

The present study addresses limitations of previous studies by providing prospective data from a Black African population, including a broad range of anomalies, and relating them to symptoms. The normative data applied to classify the anomalies were derived from the study sample with the merit of being population specific. The aim of conducting the study was to explore the association of visual efficiency anomalies and symptoms. The specific objectives addressed were: First, to determine the frequencies of (individual, categories and co-existing) refractive error, accommodative, and vergence anomalies. Secondly, determine the frequencies of symptoms and associations with anomalies in the sample studied.

## Methods

### Study design and setting

This was a cross-sectional study of patients seen in the author’s optometry practice in Empangeni, South Africa. Empangeni is a town in the uMhlathuze municipality, which is an administrative area in the uThungulu district of KwaZulu-Natal, South Africa. The city of uMhlathuze is situated on the northeast coast of the province of KwaZulu-Natal, approximately 170 kilometers northeast of Durban and borders a coastline that spans approximately 45 kilometers long.

### Sample and participants

The participants comprise consecutive patients of Black South Africans. The patients who attended the optometry practice for routine eye care come from various areas around the municipality. Analysis of the practice’s residential demography reveals that patients come from about 25 residential areas including rural, suburban and urban areas and included low- and middle-class Black persons. Included in the study were Black patients – males and females, aged between 10 and 40 years while those excluded were any patients with diabetes, ocular pathology, strabismus, or suppression. No patient record was duplicated as data for all patients including those on follow-up visit were recorded only once.

### Ethics clearance

Patients’ consents were obtained by requesting them to sign on their clinic record cards after the purpose and scope of the study was described to them. Parents or guardians consented for minors. The study protocol was approved by the Biomedical Research Ethics Committee (BE096) of the University of KwaZulu-Natal, South Africa and the conduct of the study complied with guidelines in accordance with the Declaration of Helsinki.

### Eye examination procedures

Data were collected between January and December of 2020. After demographic information were recorded, patients were sent to the data collection room. The case history entailed records of patient-reported ocular and systemic symptoms and no symptoms questionnaire was designed for this study. Referenced textbooks for the eye testing procedures included (Elliot 2021; Scheiman & Wick 2014; Grosvenor 2007; Wajuihian 2016). The preliminary tests were performed with participants wearing no correction and included visual acuity, ocular health status evaluation, suppression, and refraction. The focus of this study is on near vergence and accommodation tests therefore only near tests which were performed at 40 cm with the best refractive distance corrections in place are recorded. However, distance phoria (measured at 6 m) which is a clinical sign required to classify 3-sign convergence insufficiency was measured. Children younger than 13 years were routinely cyclopleged with a subsequent subjective refraction, which was used for analysis. Non-cyclopleged patients were fogged with + 2.00 D lens to screen for latent hyperopia. Astigmatic power and axis was refined using the Jackson cross cylinder. Given that the eye testing procedures were conventional techniques and not novel, detailed descriptions are omitted and the techniques are summarised in Table 1.

**Table 1:** Summary of data collection procedure and outcome variables.


INSTRUMENTATION/TECHNIQUE	MEASUREMENT PARAMETERS	MEASUREMENT/OUTCOME REPORTED

Case history	Patient-reported ocular-visual and systemic history.	Patient-reported eye symptoms

LogMAR charts (distance and near)	Visual Acuity	Near visual; acuity. Not reported and not relevant

Direct ophthalmoscope (Welch Allyn) & Slit lamp Biomicroscope (Zeiss SL120/130)	Ocular health	Any ocular disease excluded.

Objective refraction was done using the Autorefractor (MRK-3100; Huvitz) and the (Welch Allyn) streak retinoscope).	Distance and near refraction	

Subjective refraction with cycloplegia for younger patients’ Phoropter, cross cylinders, trial Lenses and frame as appropriate	Refined subjective refraction	Myopia, hyperopia, astigmatism, anisometropia

Royal Air Force (RAF) Rule (Birmingham Optical UK)	Near point of convergence	Near point of convergence break

Royal Air Force (RAF) Rule (Birmingham Optical UK)	Amplitude of accommodation	Reduced binocular amplitude of accommodation.

+2.00DS/-2.00DS Lens Flipper (Birmingham Optical UK)	Accommodative facility	Reduced binocular accommodative facility.

Cover test & phoropter & von Graefe	Unilateral cover test was first performed to rule out strabismus & von Graefe used to measure and quantify phoria	Distance and near phoria

Phoropter (Huvitz South korea)	Fusional vergences	Negative and positive fusional vergences

Streak retinoscope, trial lenses &frame, monocular estimation method (MEM) retinoscopy	Accuracy of accommodation	lag and lead of accommodation

Phoropter, spherical lenses	Relative accommodation	Near negative and positive relative accommodation

Worth -4- dot test (Bernell Corporation, Mishawaka Inc, USA.	Suppression assessment	Excluded patients who suppressed any eye.

Randot Stereotest (Vision Assessment Corporation USA)	Stereo-acuity	Not analyzed as it is not part of study


The focus of the study is near accommodative and vergence parameters.

### Deriving the range of normal and classifications of outcome variables

The outcome variables were refractive, accommodative, and vergence measures and symptoms. The refractive status was assigned based on the eye with the greater ametropia. Absolute values (not spherical equivalent) of refraction were used for most analysis except for anisometropia where spherical equivalent was applied. The range of normal (norms) /failure criteria was derived from the sample mean and standard deviations (SD) (Table 2). With this approach, the smaller the SD, the more the sample mean is indicative of the population mean and any measurement that falls in the range mean ± 0.7 (that is, approximately 1 SD), where 2/3 (68%) of the observations will include all values lying within one mean deviation on either side of the mean, therefore is safely within normal limits (Wajuihian 2019; Walker & Almond 2010). This approach has been used in the optometric literature (Jackson & Goss 1991; Lyon et al 2005; Scheiman et al. 2003; Scheiman et al. 1989).

**Table 2:** Frequencies of refractive error, and accommodative and vergence anomalies and associations with age gender and age.


	ALL			AGE GROUP			GENDER			
		
			10–18	19–40			MALE	FEMALE		
			
REFRACTIVE ERROR	N/%	95% CI	N/%	N/%	*χ^2^/P-VALUE*	OR 95% CI	N/%	N/%	*χ^2^/P-VALUE*	OR 95% CI

Myopia	49 (19.6)	15.91–26.72	22 (28.2)	27 (17.3)	3.10 (0.07)	1.88(0.98–3.57)	19 (21.8)	30 (20.5)	0.005 (0.94)	1.08 (0.56–2.06)

Hyperopia	75 (30.0)	26.12–38.44	22 (28.2)	53 (33.9)	0.55 (0.45)	0.76 (0.42–1.38)	29 (33.3)	46 (31.5)	0.02 (0.82)	1.08 (0.62–1.91)

Astigmatism	29 (11.6)	9.40–19.14	9 (13.2)	20 (13.9)	0.00 (0.94)	0.94 (0.40–2.19)	9 (12.0)	20 (14.8)	0.12 (0.72)	0.78 (0.34–1.82)

**Total**	153 (61.2)	51.4–84.28	53 (69.6)	100 (65.2)	3.65(1.46)	3.58 (1.8–7.14)	57 (67.1)	96 (66.8)	2.5 (0.14)	2.94 (1.52–5.79

Emmetropia	110 (44.0)	40.47–53.62	34 (43.5)	76 (48.7)	0.36 (0.54)	0.81 (0.47–1.40)	39 (44.8)	70 (47.9)	0.10 (0.73)	0.88 (0.52–1.50)

Anisometropia	9 (3.6)	1.77–7.15	5 (6.4)	4 (2.5)	1.19 (0.27)	2.6 (0.68–10.04)	3 (3.4)	6 (4.0)	0.01 (0.91)	0.84 (0.20–3.44)

**Single measure vergence anomalies**										

Near point of convergence break	33 (13.2)	10.46–20.24	18 (54.5)	15 (45.4)	7.89 (0.01)*	3.08 (1.44–6.55)	12 (36.3)	21 (63.6)	0.01 (0.91)	0.97 (0.45–2.09)

High exophoria	36 (14.4)	11.06–20.74	11 (30.5)	25 (69.4)	0.02 (0.87)	0.87(0.41–1.88)	19 (52.7)	17 (47.22)	3.74 (0.04)	2.15 (1.04–4.41)

High esophoria	15 (6.2)	3.70–10.53	5 (33.3)	10 (66.6)	0.07 (0.78)	0.99 (0.33–3.01)	4 (26.6)	11 (73.3)	0.39 (0.52)	0.59 (0.18–1.90)

Positive fusional vergence break	37 (14.8)	11.54–21.40	12 (32.4)	25 (67.5)	0.01 (0.90)	0.97(0.46–2.06)	14 (37.8)	23 (62.1)	0.00 (0.93)	1.07 (0.52–2.21)

Negative fusional vergence break	43 (17.2)	13.69–24.04	10 (23.2)	33 (76.7)	1.77 (0.18)	0.56 (0.26–1.19)	16 (37.2)	27 (62.7)	0.02 (0.82)	1.00 (0.51–1.99)

**Total**	164 (65.8)	50.45–96.95	56 (174.1)	108 (325.8)	9.765(2.74)	6.47(2.9–14.69)	65 (190.8)	99 (309.1)	4.16 (3.34)	5.78 (2.7–12.6)

**Single measure accommodative anomalies:**										

Binocular amplitude of accommodation	48 (20.0)	15.20–25.73	28 (58.33)	20 (41.6)	15.9 (0.01)*	3.84 (1.99–7.42)	14 (29.17)	34 (70.83)	0.79 (0.37)	0.69 (0.35–1.37)

Binocular accommodative facility with ± 2 lens	25 (10.2)	20.27–40.73	10 (40.0)	15 (60.0)	0.03 (0.85)	0.97 (0.37–2.53)	7 (28.0)	18 (72.0)	1.78 (0.12)	0.45 (0.16–1.23)

Lag of accommodation	13 (5.2)	2.94–9.16	11 (84.6)	2 (15.3)	14.2 (0.01)*	12.89 (2.78–59.73)	2 (15.3)	11 (84.6)	1.73 (0.10)	0.30 (0.07–1.40)

Lead of accommodation	23 (9.2)	6.23–14.15	3 (13.6)	19 (83.3)	3.18 (0.07)	0.29 (0.08–1.03)	8 (34.7)	15 (65.2)	0.00 (0.90)	0.93 (0.37–2.29)

Negative relative accommodation	76 (30.4)	25.83–37.96	43 (56.5)	33 (43.4)	27.4 (0.01)*	4.77 (2.65–8.58)	27 (35.5)	49 (64.4)	0.02 (0.80)	0.92 (0.52–1.62)

Positive relative accommodation	55 (22.0)	17.83–28.88	53 (96.3)	1 (3.6)	(0.01)*µ	353 .33 (46.6–675.2)	18 (32.7)	36 (67.2)	0.22 (0.62)	0.81 (0.43–1.54)

**Total**	240 (96.8%)	88.3 – 156.1	148 (349.5)	90(247.4)	60.90 (0.96)	376.09 (54.47–754.49)	76 (30.4)	163 (65.2)	3.75(2.82)	3.41(1.55–8.08)


*Variables with asterisk indicate significant associations.

The normative range (pass/fail criteria) are shown in Appendix A. Furthermore, accommodative and vergence anomalies were classified as single measure anomalies where there is deficiency or failure in one clinical measure (Appendix A) while anomalies with defects or failure in more than one clinical sign are described as syndrome (Appendix B). The normative range was then applied with the appropriate number of clinical signs to classify the syndromes (Appendix B).

### Symptoms

Asthenopia was defined as subjective symptoms reported by patients and in the absence of any type of ocular pathology. The symptoms of asthenopia recorded were those reported by patients. The symptoms include headaches, tearing, itchy eyes, diplopia, sandy/grittiness, photophobia, redness, tired eyes, and near blur. Furthermore, headache types were sub-classified according to the locations where the pain is mostly felt and included temporal headache (TH), frontal headache (FH), occipital headache (OH), and diffuse (DH) (non-specific). Patients’ response on intensity and duration of asthenopia was inconsistent and unreliable. Therefore, the patients enrolled included those who had consistent headaches and of the magnitude that caused much discomfort, disrupted performance of usual activities, and warranted patients consulting the optometrist. Cases of migraine headaches (confirmed by patients) were excluded as they may have different mechanism of action and headaches related to or with any manifest or underlying pathological etiology are excluded.

## Results

### Sample demographics profile

#### Age groups and gender

**Figure 1:**
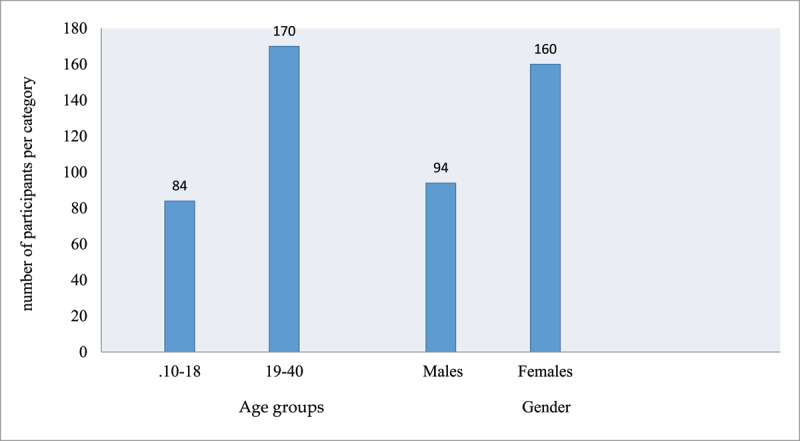
Sample demographics profile.

Only patients who met all the eligibility criteria were included in the 254 cases analysed. The age groups and gender distribution of the sample are shown in Figure 1.

### Frequency of refractive error, accommodative and vergence anomalies

#### Refractive error

The total frequency of uncorrected refractive error (URE) in the sample was 61.2%, hyperopia was the most frequent URE (30.0%) and neither age nor gender influenced the distribution of URE (Table 4).

#### Vergence anomalies

For NPC, PFV, and NFV, interest was on the break points when fusion is broken therefore recovery points were not recorded. Reduced NFV break (17.2 %) was the most frequent single vergence anomalies while 3-signs CI was the most (8.6%) for syndromes (Table 5).

#### Accommodative anomalies

Reduced NRA (30.4%) was the most frequent single measure accommodative anomaly (Table 2) while AIF (12.6%) was the most frequent for the syndrome criteria (Table 3). The frequency of 3-signs AI (AI-3) was 6 (2.3%) (Table 3).

**Table 3:** Frequencies of accommodative and vergence anomalies (syndromes) and associations with gender and age.


	ALL			AGE GROUP			GENDER			
	
			10–18	19–40			MALE	FEMALE		
			
VERGENCE ANOMALIES	N/%	95% CI	N/%	N/%	*χ^2^/P-VALUE*	OR 95% CI	N/%	N/%	*χ^2^/P-VALUE*	OR 95% CI

Convergence insufficiency (CI)	22 (8.6)	5.19–12.36	9 (10.7)	12 (7.1)	0.55 (0.46)	1.57 (0.63–3.89)	13 (13.9)	8 (5.0)	5.11 (0.01)*	3.09 (1.22–7.76)

Convergence excess (CE)	5 (1.9)	0.64–12.36	2 (2.3)	3 (1.7)	3 (0.78)	1.35 (0.22–8.24)	13 (13.9)	2 (1.2)	0.38 (0.52)	2.63 (0.43–6.06)

Fusional vergence dysfunction (FVD)	13 (5.2)	2.75 –8.59	6 (7.1)	7 (4.1)	0.51 (0.47)	1.78 (0.58–5.47)	3 (3.2)	10 (6.2)	0.57 (0.40)	0.50 (0.13–1.87)

**Total**	40 (15.7)	8.58–33.31	17(20.2)	22 (13.0)		4.7(1.43–17.6)	29 (31.0)	20 (12.4)	6.06 (0.92**﴿**	6.22

**Accommodative anomalies**										

Accommodative insufficiency (AI)	17 (6.6)	3.95–10.50	9 (10.7)	8 (4.7)	2.32 (0.12)	2.42(0.89–6.51)	5 (5.3)	12 (7.5)	0.15 (0.60)	0.70 (0.24–2.06)

Accommodative excess (AE)	5 (1.9)	0.64–4.53	2 (2.3)	3 (1.7)	1.00(.06)	1.35 (0.22–8.2)	0 (0.0)	5 (3.1)	1.57 (0.20)	0.00 (0.00–1.39)

Accommodative infacility (AIF)	32 (12.6)	8.78–17.32	16 (19.0)	16 (9.4)	3.83 (0.01)*	2.20(1.06–4.76)	10 (10.7)	22 (13.7)	0.24 (0.60)	0.76 (0.34–1.67)

**Total**	54 (21.2)	13.37–32.35	27(32.14)	27(15.98)		5.97(2.17–19.47)	15 (6.0)	39 (15.60)		1.46(0.58–5.12**﴿**


*Variables with asterisk indicate significant associations.

On demographics and outcome variables, younger patients had significantly higher frequency of receded NPC (*p* = 0.001), lag of accommodation (*p* = 0.001), reduced NRA & PRA *(p* = 0.001) (Table 2) and AIF (*p* = 0.001) (Table 3) than did older patients. Gender did not significantly impact any outcome variable.

### Frequency of coexisting refractive error, vergence, and accommodative anomalies

**Figure 2:**
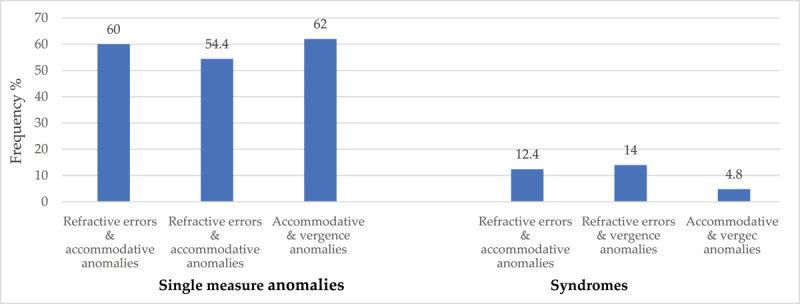
Frequencies of co-existing refractive, accommodative and vergence anomalies.

For single measure anomalies, URE and accommodative anomalies coexisted more 150 (60%) than did refractive error and vergence anomalies 136 (54.4%) (Figure 2) while accommodative and vergence anomalies coexisted most 155 (62.0%) (Figure 2).

For syndromes, URE coexisted more with vergence (14.0%) than with accommodative anomalies (12.4%) while the frequencies of coexisting vergence and accommodative syndromes was 4.8% (Figure 2).

### Frequency of symptoms

1. Overall frequency of symptomatic patients per anomaly and groups
2. The frequencies of the numbers of symptoms per patient per anomaly group

Overall, 180 (70.9%) [95% confidence interval (CI), 64.8–76.3%] of patients reported were symptomatic, while 73 (29.3) [95% CI 23.6–35.1%] reported no symptom. Headache was the most frequent symptom (41.1%); temporal headache (18.5%) was most common, while occipital headache was least frequent (Table 4). The frequency of tearing 70 (27.6%) was significantly higher in younger participants (*p* = 0.04) and photophobia 63 (24.9%). The symptom tired eye was more frequent in older participants (*p* = 0.01), while tearing was more frequent in younger patients (*p* = 0.04) (Table 4).

**Table 4:** Frequencies of symptoms for all participants and among gender and age groups.


SYMPTOMS	TOTAL (ALL)	AGE GROUP				GENDER			

		10–18 (N = 85)	19–40 (N = 169)			MALES (N = 94)	FEMALES (N = 160)		
		
		N (%)	N (%)	*χ*^2^(P-VALUE)	OR (95% CI)			*χ*^2^(P-VALUE)	OR (95% CI)

**All**	180 (70.9)	63	117			61	119		

**Headaches**						n (%)	n (%)		

Temporal	47 (18.5)	17 (20.2)	30 (17.7)			11 (11.8)	36 (22.5)		

Frontal	34 (13.4)	11 (13.1)	23 (13.6)			12 (12.9)	(13.7)		

General (diffuse)	21 (8.3)	8(9.5)	13 (7.6)	4.83 (0.30)	1.29 (0.7–2.19)	10 (10.7)	11 96.8)	5.39 (0.25)	*n/applicable*
	
Occipital	2 (0.7)	2 (2.3)	0 (0.0)			1 (1.0)	1 (0.6)		

**Total**	104 (41.1)	38 (45.2)	66(39.0)			34 (36.5)	70 (43.7)	0.9 (0.30)	0.7 (0.4–1.2)

Tearing	70 (27.6)	30 (35.7)	40 (23.6)	4.0 (0.04)*	1.7 (1.0–3.1)	24 (25.8)	46 (28.7)	0.1 (0.70)	0.8 (0.4–1.5)

Photophobia	63 (24.9)	18 (21.3)	45 (26.6)	0.5 (0.42)	07 (0.4–1.4)	21 (22.5)	41 (25.6)	0.1 (0.60)	0.8 (0.4–1.5)

Painful/sore	39 (15.4)	15 (17.8)	24 (14.2)	0.3 (0.50)	1.3(0.6–2.6)	12 (12.9)	27 (16.8)	0.4 (0.30)	0.3 (0.35–1.5)

Itchy	38 (15.2)	33 (44.0)	40(32.3)	122 (0.26)	0.6 (0.36–1.14)	28 (36.8)	47 (38.5)	0.1 (0.81)	1.0 (0.59–1.94)

Redness	34 (13.4)	14 (16.6)	20 (11.8)	0.7 (0.03)	1.4 (0.7–1.3)	13 (13.9)	21(13.1)	0.0 (0.02)	1.0 (0.5–2.2)

Tired eyes	33 (13.0)	4 (4.7)	29 (17.6)	6.5 (0.01)*	0.2 (0.08–0.71)	8 (8.6)	25 (15.6)	1.8 (0.12)	0.5 (0.2–1.1)

Grittiness	14 (5.5)	2 (2.3)	12 (7.1)	1.5 (0.22)	0.3 (0.0–1.4)	5 (5.3)	9 (5.6)	0.0 (0.80)	0.9 (0.3–2.9)

Near blur	8 (3.1)	2 (2.3)	6 (3.5)	0.0 (0.90)	0.6 (0.1 –3.3)	4 (4.3)	4 (2.5)	0.1 (0.60)	1.7 (0.4–2.9)

Diplopia	6 (2.3)	3 (3.5)	3 (1.7)	0.2 (0.60)	2.0 (0.4–10.3)	4 (4.3)	2 (1.5)	1.2 (0.20)	3.5 (0.6–19.7)


*Variables with asterisk indicate significant associations.

### Symptoms and anomalies

#### Overall symptoms & anomalies

For refractive error, patients with low astigmatism (44.8%) were most symptomatic, emmetropia [(defined as ± 0.25 /low power spherical errors) (33.6%)] hyperopia was 22%, while astigmatism (≥ 0.75 diopter cylinder) was least symptomatic (9.2%) (Appendix C & Table 5, column 2).

**Table 5:** Frequency of symptoms and associations with anomalies.


VARIABLES	SYMPTOMATIC PARTICIPANTS		1 SYMPTOM	2 SYMPTOMS	3 OR MORE SYMPTOMS	NO SYMPTOMS	TOTAL	*χ*^2^(P-VALUE)
	
GENDER		*χ*^2^(P-VALUE)	N (%)	N (%)	N (%)	N (%)	N (%)	

Male	61 (24.4)	1.52 (0.01)	15 (6.2)	12 (4.8)	34 (13.6)	33 (12.0)	94 (37.2)	0.009 (0.92
	
Female	118 (47.2)		27 (10.8)	34 (13.6)	57 (22.8)	42 (15.4)	160 (62.8)	
	
**Total**	179 (72.6)		42 (17.0)	46 (18.4)	91(36.4)	75 (27.4)	254 (100)	

**Age (years)**								

10–18	70 (28)	8.23 (0.01)*	20 (8.0)	17 (6.8)	33 (13.2)	14 (5.6)	84 (33.6)	10.59 (0.01)*
	
19–40	110 (44)		23 (9.2)	29 (11.6)	58 (23.2)	59 (23.6)	169 (67.6)	
	
**Total**	180 (72.0)		43 (17.2)	46 (184.)	91 (36.4)	73 (29.2)	254 (100)	

**Anomalies**								

**Refractive error**								

Myopia	41 (16.4)	1.88 (0.39)	15 (6.0)	10 (4)	16 (6.4)	8 (3.2)	49 (19.6)	7.31 (0.29)
	
Hyperopia	55 (22.0)		9 (3.6)	18 (7.2)	28 (11.2)	20 (8)	75 (30)	
	
Astigmatism	23 (9.2)		7 (2.8)	7 (2.8)	9 (3.6)	6 (2.4)	29 (11.6)	

**Total**	119 (47.6)		31(12.0)	35(14)	53 (21)	30 (12.0)	153 (61.2)	

Emmetropia (0 to ± 0.25 DS)	84 (33.6)	19.96(0.01)*	19 (7.6)	18 (7.2)	47 (18.8)	21 (8.4)	105 (42.0)	

Low astigmatism (0.25–0.5) DC	112 (44.8)		35 (217)	37 (23.0)	71 (44.1)	24 (10.0)	136 (54.4)	

Anisometropia		0.17(0.61)	5 (2.0)	1 (0.4)	0 (0)	3 (1.2)	9 (3.6)	

**Syndromes**								

**Vergence anomalies**								

Convergence insufficiency	16 (6.4)	3.85 (0.41)	3 (1.2)	2 (0.8)	11 (4.4)	5 (2)	21 (8.4)	5.45(0.48)
	
Convergence excess	2 (0.8)		1 (0.4)	0 (0)	1 (0.4)	3 (1.2)	5 (2.0)	
	
Fusional vergence dysfunction	11 (4.4)		1 (0.4)	2 (0.8)	8 (3.2)	2 (0.8)	13 (5.2)	

**Total**	26 (11.6)		4 (2.0)	4 (2.0)	20 (8.0)	10(4.0)	39 (15.6)	

**Accommodative anomalies**								

Accommodative insufficiency	13 (5.2)	0.50 (0.78)	2 (0.8)	2 (0.8)	9 (3.6)	4 (1.6)	17 (6.8)	1.27(0.97)
	
Accommodative excess	4 (1.6)		1 (0.4)	1 (0.4)	2 (0.8)	1 (0.4)	5 (2.0)	
	
Accommodative infacility	22 (8.8)		5 (2.0)	3 (1.2)	14 (5.6)	10 (4.0)	32 (12.8)	
	
**Total**	39 (15.6)		8 (3.0)	6 (2.4)	25 (10.0)	15(6.4)	54 (21.6)	

**Single measure anomalies**								

**Vergence anomalies**								

Receded near point of convergence break	28 (11.2)	4.20 (0.52)	7 (2.8)	7 (2.8)	14 (5.6)	5 (2.0)	33 (13.2)	9.05 (0.87)
	
High exophoria	28 (11.2)		8 (3.2)	3 (1.2)	17 (6.8)	8 (3.2)	36 (14.4)	
	
High esophoria	9 (3.6)		2 (0.8)	2 (0.8)	5 (2.0)	6 (2.4)	15 (6.2)	
	
Reduced positive fusional vergence break	29 (11.6)		5 (2)	5 (2)	19 (7.6)	8 (3.2)	37 (14.8)	
	
Reduced negative fusional vergence break	34 (13.6)		9 (3.6)	9 (3.6)	16 (6.4)	9 (3.6)	43 (17.2)	
	
Total	128 (51.2)		31(12.0)	26 (10.0)	71 (28)	36 (14.4)	164 (65.6)	

**Accommodative anomalies**								

Reduced binocular amplitude of accommodation	29 (11.6)	10.5 (0.06)	6 (2.4)	4 (1.6)	19 (7.6)	7 (2.8)	36 (14.4)	21.97 (0.10)
	
Reduced binocular accommodative facility	20 (8.0)		3 (1.2)	9 (3.6)	8 (3.2)	5 (2)	25 (10.0)	
	
High Lag of accommodation	12 (4.8)		2 (0.8)	5 (2.0)	5 (2.0)	1 (0.4)	13 (5.2)	
	
High lead of accommodation	8 (3.2)		1 (0.4)	1 (0.4)	6 (2.4)	5 (2.4)	13 (5.2)	
	
Negative relative accommodation	59 (23.6)		9 (3.6)	10 (4.0)	29 (11.6)	17 (6.8)	76 (30.4)	
	
Positive relative accommodation	30 (12.2)		17 (10.8)	13 (5.2)	20 (8.0)	25 (10.0)	55 (22.0)	
	
Total	158 (63.2)		38 (15.2)	42 (16.8)	87 (34.8)	60 (24.0)	218 (87.2)	


*Variables with asterisk indicate significant associations. Table format was adapted from Hashemi et al. 2019.

For single measure vergence anomalies, patients with NFV 34 (13.6%) were most symptomatic while reduced NRA 59 (23.6%) was most symptomatic for accommodative anomalies (Table 5). For the accommodative-vergence syndrome, AIF (8.8%) and CI (6.4%) were most symptomatic (Table 5 column 2).

#### Frequencies of number of symptoms and anomalies

For three or more symptoms patients with low-amount astigmatism (44.1%) emmetropia (18.8%) and hyperopia (11.2%) had the highest frequency of number of symptoms.

For single measure anomalies, participants with reduced NRA (30.4%), PRA (22%), and NFV (13.6%), had higher frequency of number of symptoms. For syndromes, patients with AIF (5.6%), CI (4.4%), and AI (3.6%) presented the highest frequency of symptoms.

**Figure 3:**
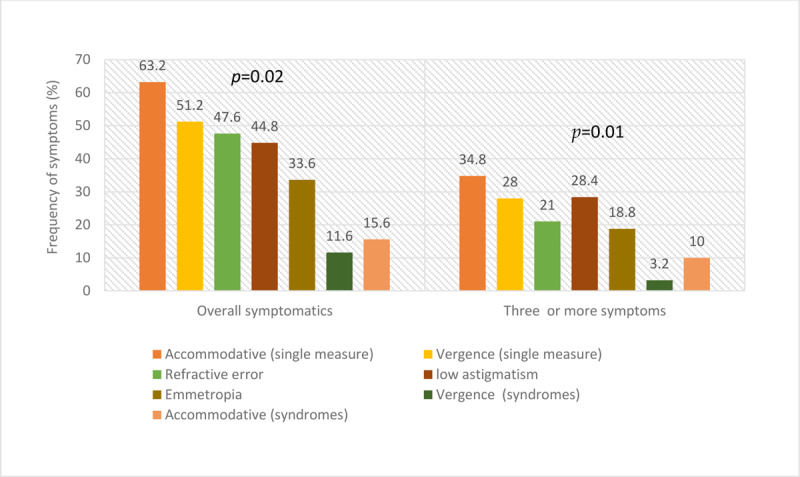
Frequency of refractive error, accommodative and vergence anomalies.

Overall, based on number of symptomatic, number of symptoms per anomaly and associations with specific symptoms, patients with accommodative anomalies were more symptomatic compared to vergences anomalies and refractive errors (Table 5 and Figure 3). The distribution of overall symptoms and of the number of symptoms is similar (Table 5). It is also noteworthy that although patients with NRA and NFV showed higher frequencies of symptoms generally (Table 5), they were less specific to individual symptoms (Appendix D).

A summary of the first three anomalies within each anomalies category most associated with each symptom type is presented in Appendix E. For headaches, emmetropia, low astigmatism and hyperopia had the highest frequencies of headaches, reduced amplitude of accommodation, exophoria and reduced PFV while AIF, AI, and CI had the highest frequencies of symptoms for single measures and syndromes categories respectively. Accommodative anomalies were most symptomatic (Figure 3).

## Discussion

This study reported the frequencies of and associations among visual efficiency anomalies and symptoms in patients aged between 10 and 40 years examined at an independent optometry practice. For syndromes, accommodation anomalies (21.2%) were more frequent than vergence anomalies (15.2%). Furthermore, hyperopia (30.0%), AIF (12.6%), and CI (8.6%) were the most frequent anomalies for each category respectively. On the frequencies of association, refractive error coexisted more with accommodative than with vergence anomalies, whereas accommodative and vergence anomalies co-existed most. Regarding symptoms, 70.9% of patients studied were symptomatic, headache (41.1%) was the most frequent symptom type, and females and older patients had significantly higher frequency of symptoms. Regarding frequencies of anomalies and symptoms, emmetropia, low-magnitude astigmatism and hyperopia, reduced amplitude of accommodation, CI, and AIF were most frequently associated with symptoms of asthenopia. Overall, the high frequencies of visual efficiency anomalies suggest an increased awareness and uptake of optometric care for near vision anomalies in the population. The high frequency of symptoms suggests that majority of patients consulted the optometrist because they experienced bothersome symptoms which are related to visual efficiency anomalies.

### Frequencies and associations among anomalies

The 61.2% frequency of total uncorrected refractive error is comparable to findings from other studies, which ranged between 62–69.9% (McKay, Woodruff & Rumsey 2002; Ngakhushi et al. 2018; Scheiman et al 1996; Vaishali, Jha & Srikanth 2019). The ways the anomalies are classified are important in clinical practice and epidemiology. A study by Rouse et al. (1997) highlighted this relevance of classifying anomalies based on number of clinical signs (single or multiple). The authors found that 93% of practitioners applied the single NPC criterion, 80% PFV criteria (60.5% Morgan’s data (Morgan 1944)) or Optometric Extension Programme (OEP) norms (Scheiman & Wick 2014) and Sheard’s criterion 3.5% while 75% used near phoria. On the number of clinical signs used to diagnose CI, Rouse et al. (1997) found that 35% of practitioners favoured single criteria while 49% preferred using multiple criteria to define CI. Although the findings by Rouse et al. (1997) remain relevant to research and clinical practice, the approach is often ignored. In the present study, both criteria (Appendix A & B) were applied to classify anomalies, whereas most available studies (Appendix F) applied only the single or syndrome criteria.

An unexpected finding in the present study is a greater frequency of reduced AA among younger patients. [58.3% versus 41.6%, *p* = 0.001] although the mean AA was lower for older patients (*p* = 0.01); therefore, difference between the findings on means and frequencies may be related to cut-off used to define frequencies. However, such unusually reduced AA may be of interest given that two studies on primary school populations in South Africa (Metsing & Ferreira 2012; Moodley 2008) reported similar trends of greater frequencies of reduced AA among younger patients. In Sweden, Sterner et al. (2004) found a significantly lower mean AA among younger primary school children and concluded that the AA of children may not be as good as expected. These reports of poorer accommodative amplitude among younger participants may be a unique variation in populations which will require further investigations.

For syndromes anomalies, AIF-12.6% and CI-8.6% were the most frequent anomalies (Table 3). For high school children aged between 13 and 18 years, South Africa, Wajuihian and Hansraj (2016) found AIF (12.9%) to be the most prevalent accommodative anomaly. Moodley (2008) found that 30% of a sample of school children in South Africa failed the AF test (single measure anomaly). The relatively high frequency of AIF in the present study may be related to the fact that most patients with accommodation difficulties manifest AIF and show both patterns of inadequate responses to stimulation (accommodative insufficiency) and relaxation (accommodative excess) (Garzia 2006).

CI is a prevalent and commonly reported vergence anomaly and 8.6% of patients in the present study had CI. In a school-based study in South Africa, Wajuihian and Hansraj (2016) found the prevalence of CI for children aged 13 and 18 years to be 10.3%, which is comparable to 10.7% frequency for age group 10 to 18 years in the present study. This analogy is noteworthy given that the study by Wajuihian and Hansraj (2016), and the present study were conducted with participants from the same geographic area, using same classification criteria. Similarities of the findings suggest a consistent prevalence estimates in these populations regardless of the different study settings (clinical versus non-clinical). Furthermore, compared to previous studies, 8.6% frequency of CI in the present study is greater than 7.7% prevalence reported by Ovenseri-Ogbomo and Eguegbu (2016). The study by Ovenseri-Ogbomo and Eguegbu (2016) is the only available study on African population (Appendix F), although their sample were first-year university students who presented for pre-university entry vision screening.

Regarding previous studies on non-African populations, the findings from present study are more comparable to some studies on independent optometry practice especially for CI (Appendix F). The difference in findings across studies (Appendix F) is related to a lack of validated and standardized protocol for testing and classifying anomalies. Inconsistent findings remain a major concern in studies on vision efficiency anomalies as it makes it difficult to derive a consistent prevalence estimates for policy administration in planning. The Convergence Insufficiency and Reading Study Group (CIRS) (Rouse et al. 1998) is the only available standardized testing protocol, which was specific for CI. Another important aspect of the research discourse worth elaborating on is the criteria applied to classify the anomalies, as well as how the range of normal (norms) for the criteria are derived. Using the normative data derived from study sample as used in the present study is likely to improve study validity. Several studies (Appendix F) used non-sample-specific norms such as the Morgan’s data and the CIRS system, which were derived from a broad range of patients.

On co-existing anomalies, refractive error coexisted more with accommodative (60%) than vergence anomalies (54.4%) (Figure 2). Dwyer and Wick (1995) found that 64% of patients had refractive errors coexisting with vergence and accommodative anomalies. Accommodative and vergence coexisted most at 62% for single measure anomalies and 4.8% for syndromes. Findings from other studies ranged between 2.8 and 3.8% (García-Muñoz et al. 2016; Hoseini-Yazdi et al. 2015; Mandal & Kamathet 2020; Moon, Kim, &Yu 2020). Understanding coexisting anomalies will enable the clinician to identify anomalies, which may have unique symptoms. Such information will guide towards targeted treatment.

### Frequency of symptoms

Overall, the high frequency of symptomatic patients (70.9 %) in the present study agrees with other studies (Daum 1983; Neugebauer, Fricke & Rußmann 1992; Mvitu & Kaimbo 2003; Vaishali, Jha & Srikanth 2019). In the study by Montes-Mico (2001), 63% of patients were symptomatic, and Hashemi et al. (2019) found 70.9% of university students in Iran had asthenopia which is the same as present study. Headaches were the most frequent symptoms, which is similar to reports from other studies (Appendix G). Ocular headache of functional cause is an acute or chronic discomfort that results from prolonged performance of near task. Ocular headache is a reflex (referred) pain, which results from stimulation of the endings of the nasal branch of the ophthalmic division of the fifth cranial nerve (Cameron 1976; Wajuihian 2015).

### Associations between Symptoms, refractive error, accommodative and vergence anomalies

Emmetropia, low-amount astigmatism (LA) and hyperopia were the refractive errors most with symptoms. Clinical experience, findings from the present study and narrative reports, (Bellow 1968; Grosvenor 2007) indicate that lower spherical and cylindrical errors tend to cause more symptoms than high amount errors although empirical studies to support the report could not be found. In general, refractive errors may produce headaches and pains in the frontal, bi-temporal and occipital regions and at the back of the neck (Cameron 1976). Furthermore, LA has been hypothesized to cause changes to visual perception that alter the hyperexcitability in the visual cortex of the brain of headache sufferers (Marasini et al. 2012). Furthermore, in astigmatic patients, the presence of symptoms depends on factors, which include amount of astigmatism present (Grosvenor 2007; O’Leary et al. 2003). With high astigmatism, the ciliary muscles may make minimal effort to correct the error and there may be asthenopia (Grosvenor 2007; Wajuihian 2015). However, if the degree of astigmatism is low or moderate, patients make unconscious efforts to compensate for the error. This is sometimes overdone, causing the ciliary muscle to contract irregularly, thus results in more asthenopia.

Hyperopia is frequent and has consistently been associated with asthenopia (Junghans et al. 2020; Rydberg 2005) and reduced reading ability, impaired performance on visual perception as well as poor school performance (Grosvenor 2007). Even a low-amount hyperopia may cause other symptoms including intermittent blur, fatigue, loss of concentration and inattention in some children, which may be mistaken for a short attention span (Grosvenor 2007; Wajuihian 2015). High frequencies of symptoms in uncompensated hyperopia are often related to the excessive accommodative demand in hyperopia. In myopia, symptoms are varied and in addition to impaired distance vision, myopes attempt to see clearer and squint to cut off diffusion circles by narrowing the palpebral fissure therefore manifest symptoms (Grosvenor 2007).

Regarding symptoms and accommodative and vergence anomalies, patients with accommodative anomalies were more symptomatic than vergence anomalies. For single measure anomalies, NFV was notably symptomatic. This finding may be considered unexpected although Borsting et al. (2003) and Sedaghat and Abrishami (2014) found significant association between NFV and symptoms. Significant association between NFV and symptoms suggest a power of compensation of larger amounts of base-in prisms (Sedaghat & Abrishami 2014). This hypothesis requires further investigation. Within syndromes, AA, AIF, and CI were significantly most associated with symptoms (Table 5). Findings on impaired accommodative facility and symptoms seem consistent in the literature (Hennessey, Iosue & Rouse 1984; Iribarren, Fornaciari & Hung 2001). Accommodative facility is considered the clinical measure most likely to predict symptoms (Hennessey, Iosue & Rouse 1984) given that AF testing and function is a dynamic test, more interactive, and forces the accommodative-vergence to respond to frequent changes in the stimulus (Garzia & Nicholson 1991; Hennessey, Iosue & Rouse 1984). High symptom rates in defective AF may be substantial given that most patients with accommodation difficulties manifest AIF, which involves both patterns of inadequate responses to stimulation (accommodative insufficiency) and relaxation (accommodative excess) (Garzia 2006). Therefore, the AF may be the most engaged accommodative function in a classroom setting and more prone to defects. High symptom frequency report in reduced AA is related to the continued effort of the accommodative mechanism in focusing and refocusing (Garzia 2006).

For vergence anomalies, patients with CI were most symptomatic (26.4%) and the significant association between CI and frontal headaches corroborates the findings by Marasini et al. (2012) and Priya et al. (2021). The 26.4% frequency of symptoms in CI from the present study is lower than reports from other studies (Daum 1984; Horwood, Toor & Riddell 2014; Rouse et al. 1998; Vaishali, Jha & Srikanth 2019). Difference in findings across studies may be related to study designs especially the use of different classification criteria.

Being a functional anomaly, asthenopia may manifest mainly when prolonged intense near point activates are performed. Invariably, report of symptoms is likely to be higher for studies on clinical populations where the major reasons for attending the optometrist may be due to existing symptoms. Therefore, snap short testing and recording as is done during vision screening may not identify the symptomatic cases. Furthermore, signs in CI for example can exist for years without any symptoms, which may disappear entirely while the signs remain unaltered (Wajuihian & Hansraj 2016). In general, asthenopia is in most cases, manifestations of anomalies in the visual efficiency system and can be relieved by commencing with compensating for the baseline refractive errors and any co-existing anomalies.

### Implications, applications, limitations and strengths

The present study advances knowledge as it provides data on a broad range of visual efficiency anomalies in relation to symptoms in an independent optometry clinic, which has not been studied previously. This study highlights the need for optometrists to routinely test for accommodative and vergence anomalies and to consider the coexistence of anomalies in relation to symptoms before treatments are initiated. This approach will enable appropriate treatment for anomalies.

The clinical setting of the study, the lack of random sampling and the relatively small sample size limits the external validity of this study. Therefore, the findings should be applied and interpreted in the context of the study design, which includes being clinic-based and the participants’ age range. However, the study has several strengths, which include its relatively firm prospective design, its novelty, and the heterogeneous nature of the patient base, which comprised low- and middle-class Black persons who resided in several rural and suburban areas. Using population-specific norms and documenting both single signs and syndrome disorders to define anomalies improves identifying and reporting on coexisting anomalies and increases the applicability of findings for research and clinical practice. This study has implications and applications for clinical practice and research in vision care and will guide future studies on differential diagnoses.

This issue of deriving norms for sample as well as, criteria and definition criteria require continued discourse. Further studies using larger sample sizes and prospective and random sampling could yield a more generalisable finding. Obtaining reliable prevalence estimates is important for epidemiology. Further studies are needed to investigate which symptoms are unique to each anomaly. Standardised study protocols and symptoms surveys for various study settings will be useful to improve study validity and prevalence estimates.

## Conclusion

For this clinical patient sample aged 10–40 years old, refractive, accommodative, and vergence anomalies and symptoms were frequent. Accommodative anomalies were more frequent than vergence. The frequency of coexisting accommodative and vergence anomalies was high and refractive errors and accommodative anomalies coexisted more frequently than refractive errors and vergence anomalies. Furthermore, uncorrected refractive error most frequently coexisted with accommodative and vergence anomalies than did accommodative and vergence anomalies.

Headache was the most frequent symptom. Low spherical and cylindrical errors were the most frequent refractive source of asthenopia whereas accommodative infacility and CI were most frequently associated with symptoms.

## Additional File

The additional file for this article can be found as follows:

10.22599/bioj.267.s1 Appendices. Appendix A to G.
