# Supplementary material for: Characterizing Refractive Errors, Near Accommodative and Vergence Anomalies and Symptoms in an Optometry Clinic
Source: Br Ir Orthopt J. 2022 Jul 14;18(1):76–92. doi: 10.22599/bioj.267 (PMC9284986; doi:10.22599/bioj.267)
Supplement: Appendices. — Appendix A to G. [file bioj-18-1-267-s1.pdf]

APPENDIX

APPENDIX A: Failure criteria for clinical signs for outcome variables

Clinical measures	Failure cut-off criteria and age groups	
**a) Refractive error (unit)**	**10–40 years (All)**	
Myopia (DS)	≥ (□) 0.5 DS	
Hyperopia (DS)	≥ 0.50 DS	
Astigmatism (DC)	≥ - 0.75 DC	
Emmetropia (DS)	+/-0.50	
Anisometropia (DS)	Difference of ≥ 0.75 or - 0.75 DC between Right and left eye)	
**b) Accommodative measures (abbreviations/unit)**	**10–18 years**	**19–40 years**
Reduced binocular amplitude of accommodation binocular (AA) (D)	≤ 12	≤ 9
Reduced binocular accommodative facility (AF) using +2.00DS/-2.00DS flipper lenses (cpm)	≤ 7	≤ 6
Lag (D)	> + 0.75	> +1.00

Lead (D)	< + 0.25	0.25
Reduced negative relative accommodation (NRA) (DS)	≤ 2	≤ 1.50
Reduced positive relative accommodation (PRA) (DS)	≤ -2.50	≤ 1.00
**c) Vergence measures(unit/abbreviations)**	**10-18 years**	**19-40 years**
Near point of convergence break (NPC) (cm)	≥ 8	≥ 10
Distance phoria (pd)	0	0
Near exophoria (pd)	≥ 6	≥5
Near esophoria (pd)	≥ 2.50	≥ 1.00
Negative fusional vergence break (NFV) pd	≤ 11	≤ 12
Positive fusional vergence break (PFV) pd	≤ 12	≤ 13

APPENDIX B Criteria used to classify syndrome anomalies

Syndromes	Clinical signs
**Vergence syndromes**	
a) Convergence insufficiency	All 3 signs<br>(1) Exophoria at near) greater than far at least 6pd<br>(2) Receded NPC<br>.<br>(2) Insufficient fusional vergence: (i) fails Sheard's criteria or (ii) reduced PFV at near.
b) Convergence excess	Minimum of 2 clinical signs<br>(1) Significant esophoria at near<br>(2) Reduced NFV break at near /break<br>(3) High MEM
c) Fusional vergence dysfunction	Reduced fusional vergence range (PFV & NFV (2) Orthophoria, (3) minimal refractive error.
**Accommodative syndromes**	
d) Accommodative insufficiency	Minimum of clinical signs 1 and 2 or 1 and 3 or all clinical signs<br>(1) Reduced AA. Push-up monocular AA at least 2 D below Hofstetter's calculation for minimum amplitude: 15 – 0.25 X age (years)<br>(2) High values on monocular estimation retinoscopy<br>(3) Fails monocular AF testing.
e) Accommodative excess	Clinical signs (1) and (2) or (1) and (3)<br>(1) Low MEM<br>(2) Difficulty clearing + 2.00 D with monocular accommodative facility with criterion.<br>(3) Fails binocular accommodative facility test with + 2 .00 D
f) Accommodative infacility	Clinical sign (1) and (2) or (1) and (3)<br>1) Fails binocular and monocular accommodative facility using ± 2.00 D lenses.<br>2) Reduced positive relative accommodation PRA used acronym below, why not here too?<br>3) Reduced NRA

APPENDIX C: Symptoms and anomalies

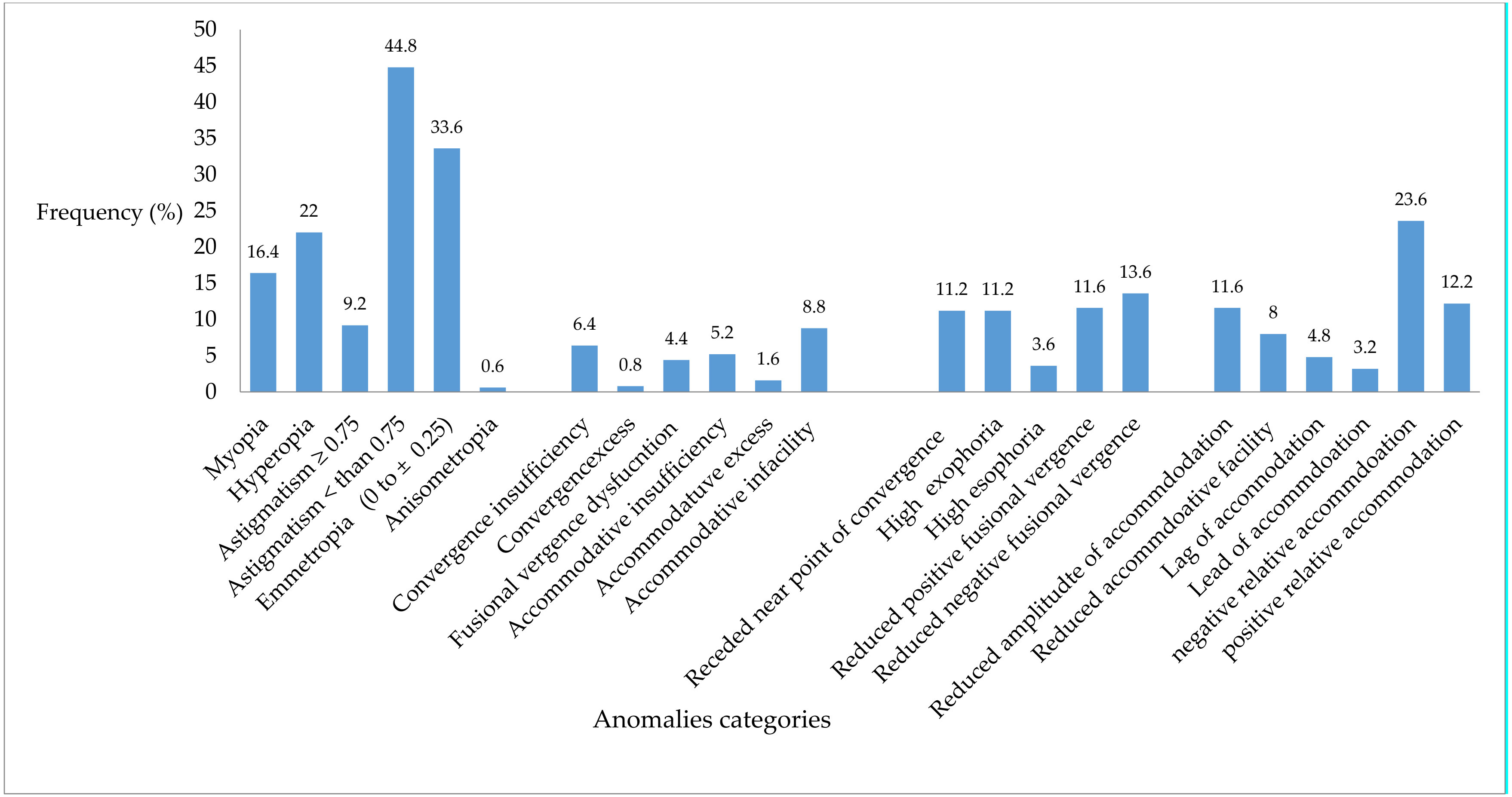

APPENDIX D: Associations among specific symptoms and anomalies

**Anomalies and symptoms**	**Yes, responses n/%**	**χ^2 and *p*-values**	**Bivariate logistic regression. /OR 95%, confidence interval**
Fusional vergence dysfunction & painful eyes	5 (12.8)	3.9 (0.05)	3.8 [1.1-12.3]
Convergence insufficiency & photophobia	11 (17.4)	**7.7 (0.01)**	3.8 [1.5-9.5]
Accommodative insufficiency & tired eyes	33 (13.2)	0.05*µ	[1.31-45.8]
Reduced amplitude of accommodation & tired eyes	18 (60.0)	5.5 **(0.02)**	2.7 [1.24-5.9]
Lead of accommodation & diplopia	2 (33.3)	0.04 Fischer test	10.05 [1.6-60.9]
High exophoria & grittiness	16 (25.4)	**6.21 (0.01)**	2.71 [1.2-5.6]
Positive fusional vergence & painful eyes	12 (30.7)	**6.33 (0.01)**	*2.4* [1.18-5.09]
Exophoria & grittiness	2 (14.2)	**6.20 (0.01)**	2.7 [1.2-5.6]
Myopia	4 (10.5)	**9.04 (0.01)**	N/A

*Variables highlighted in bold indicate significant associations.

APPENDIX E: Symptoms with three anomalies most assoiated with them.

**Symptom types**	**Refractive error**			**Single measure anomalies**			**Syndromes**		
Headache	Emmetropia	Low astigmatism	hyperopia	Reduced amplitude of accommodation	exophoria	Reduced PFV	AIF	CI	AI
Tearing	Emmetropia	Low astigmatism	hyperopia	Reduced amplitude of accommodation	exophoria	Reduced PFV			
Tired eyes	Emmetropia	Low astigmatism	hyperopia	Reduced amplitude of accommodation	exophoria	Reduced PFV	CI	AE	
Painful	Emmetropia	Low astigmatism	hyperopia	Reduced amplitude of accommodation	rReduced PFV	Receded NPC	CI	AIF	
Itchy	Emmetropia	Low astigmatism	hyperopia	Reduced amplitude of accommodation	rReduced PFV	Receded NPC	CI	AI	AE
Photophobia	Emmetropia	Low astigmatism	hHyperopia & myopia	Reduced amplitude of accommodation	eExophoria	Reduced PFV	AIF	CI	
Grittiness	Emmetropia	Low astigmatism	hyperopia						
Diplopia	Myopia	-	-	Reduced amplitude of accommodation	rReceded NPC	Reduced PFV	AI	AIF	
Near blur	Low astigmatism	hyperopia		Reduced amplitude of accommodation	rReduced PFV				
Redness	Low astigmatism	myopia		Reduced PFV			AIF	FVD	AI

PFV (positive fusional vergence), NPC (near point of cocnvergence),CI (convergence insufficiency), AI (accommodative insufficiency) AIF (accommodative infacility),AE (accommodative excess), FVD (fusional vergence dysfunction)
Blank spaces indicate not applicable

APPENDIX F: Frequencies of accommodative and vergence anomalies from previous studies

Study setting	Country of study	Age (years)	Sample size	AI	AE	AIF		CI	CE	FVD	
**Independent optometry practice (IOP)**				**Syndrome**							
Ma et al. (2019)	China	21–38	415	8.4	-	2.1		9.6	1.1	5.3	
Hokoda (1985)	USA	22.9 ***	119	9.2	2.5	5.1		4.2	-	-	
Lara et al. (2001)	Spain	10–35	265	3	6.4			0.8	4.5	-	
Montes-Mico (1985)	Spain	18–38	1679	11.4	6.5	10.3		5.9	1.5	1.8	
Skjöld et al. (2018)	Sweden	8–35	159	17.6	N/A	N/A		9.4		1.5	
**University optometry clinic (UOC)**											
Ovenseri-Ogbomo & Eguegu (2016)	Nigeria	15–28	212	N/A	N/A	N/A		7.7	1.5	1.5	
Mckay, Woodruff & Rumsey (2002)	USA	10–38	158	15.5	N/A	8.6		7.8			
Paniccia et al. (2015)	Puerto Rica	5–20	593	39	5.1	7.6		12.6	9.1	4.7	
Moon et al. (2020)	S/Korea	18–28	184	3.8	1.1	N/A		18.5	2.2	-	
Hoseini-Yazdi et al. (2015)	Iran	21.3 ± 3.5	83	2.4	4.8			4.8	4.8	-	
Richman & Laudon (2002)	USA	24–31	48	N/A	N/A	N/A		13	8	6	
Porcar-Martinez (1997)	Spain	22 ***	65	6.2	10.8	N/A		7.7	-	-	-
Rouse et al. (1998)	USA	8–12	415					6.0			
**Hospital eye clinic (HEC)**											
Marasini et al. (2012)	Nepal	17–40	100					16.2	N/A	11.2	-
Mandal1 & Kamath (2020)	India		161	3.2	0.6	7.7		7.7	0.6	7.0	-
Vashali, Jha & Srikanth (2019)	India	18–35	142					27.4	-	12.7	-
Magdelene et al. (2017)	India	10–40	131	9.4	2.0	5.1		32	6	2.2	-
Ngakhushi et al. (2018)	Nepal	19–59	103	41. 3	1.0	40. 0		61. 2	-	-	-

Single measures anomalies

	Country of study	Age (years)	Sample size	AA	AF	NRA	PRA	NPC break	Exophoria	NFV	PFV
Skjöld et al. (2018) (Independent optometry practice)	Sweden	8–35	159		58.5	28.9	62.3		77.4	60.4	52.8

Ngakhushi et al. (2018) (Hospital eye clinic)	Nepal	19–59	103	Lag, 30.4, lead 4.3	11.6	11. 7		50.0	89.3	36. 9

APPENDIX G: Summary of previous studies on symptoms

	Present study (2022)	Ngakhushi et al. (2018)	Neugebauer, Fricke & Rußmann (1992)	Mohamed doud Ali (2017)	Mvitu & Kaimbo (2003)	Dwyer (1992)	Alexander et al. (1985)	Westman et al. (2012)	Daum (1984)	Daum (1983)
**Symptoms**										
Headaches	41.1	63.1	84	93	47.0	15	11.6	62.7	54	56
Tearing	27.6		–		24.5	–	1.2	3		
Ocular pains	15.4	67. 6	34		37	–	2	14.8		
Distance blur/ Blurry vision	18.1	35. 9		90					47	59
Near blur	3.1	15. 5	–	70	–	23	7.6	69.6	19	9
Tired eye	13.0									
Red eye	13.4		44		4.3	–	0.8			
Photophobia	24.9		48		20		2		3	3
Diplopia	2.3	9. 71	10		4.3	1	0.5	21.5	47	30
Grittiness	5.5									
Burning sensation			–	13	12.5	–				
			–		–	–	1			

References

Alexander, JA, Joyes, D, Liew, C, Omrod, M and **Wong, A.** 1985. A survey of cases in Sydney optometric practices: Age and sex distributions; symptoms and reasons for consultation. *Aust J Optom*, 68: 133–138. DOI: https://doi.org/10.1111/j.1444-0938.1985.tb01075.x

Magdalene, D, Dutta, P, Choudhury, M, Deshmukh, S and **Gupta, K.** 2017. Clinical profile of non-strabismic binocular vision anomalies in patients with asthenopia in North-East India. *TNOA J Ophthalmic Sci Res*, 55(3): 182–186. DOI: https://doi.org/10.4103/tjosr.tjosr_36_17

Porcar, E and **Martinez-Palomera, A.** 1997. Prevalence of general binocular dysfunctions in a population of university students. *Optom Vis Sci*, 74(2): 111–113. DOI: https://doi.org/10.1097/00006324-199702000-00023

Rouse, MW, Hyman, L and **Hussein, M.** 1998. Frequency of convergence insufficiency in optometry clinic settings. Convergence Insufficiency and Reading Study (CIRS) Group. *Optom Vis Sci*, 75(2): 88–96. DOI: https://doi.org/10.1097/00006324-199802000-00012

Rouse, MW, Hyman, L and **Hussein, M.** 1998. Frequency of convergence insufficiency in optometry clinic settings. Convergence Insufficiency and Reading Study (CIRS) Group. *Optom Vis Sci*, 75(9): 88–96. DOI: https://doi.org/10.1097/00006324-199802000-00012

Skjöld, G, Malmö, SA, Brinkby, G and **Cheng, Y.** 2018. Binocular measurements in a non-selected group of non-strabismic patients 8–35 years old, in Sweden. *Optometry & Visual Performance*, 6(1): 31–37.

Mohamed, AM. 2017. Frequency of presenting clinical features of asthenopia (ocular fatigue) in refractive patients. *Opthalmology Pakistan*, 7(3): 15–19.
